# Photo-crosslinked adhesive hydrogel loaded with extracellular vesicles promoting hemostasis and liver regeneration

**DOI:** 10.3389/fbioe.2023.1170212

**Published:** 2023-05-10

**Authors:** Wuzheng Xia, Guanzhi Lai, Yichuan Li, Cong Zeng, Chengjun Sun, Pinzhe Zhang, Guanghao Zhu, Leping Li, Linwei Wu

**Affiliations:** ^1^ Department of Organ Transplantation, Guangdong Provincial People’s Hospital, Guangdong Academy of Medical Sciences, Southern Medical University, Guangzhou, China; ^2^ Shantou University Medical College, Shantou, China; ^3^ Department of Hepatobiliary and Pancreatic Surgery, People’s Hospital of Guang’an City, West China-Guang’an Hospital, Sichuan University, Guang’an, China; ^4^ Guangdong Provincial Key Laboratory of Gastroenterology, Department of Gastroenterology, Nanfang Hospital, Southern Medical University, Guangzhou, China; ^5^ Department of General Practice, Hospital of South China Normal University, Guangzhou, China; ^6^ The Second School of Clinical Medicine, Southern Medical University, Guangzhou, China

**Keywords:** adhesive hydrogel, extracellular vesicles, hemostasis, liver regeneration, tissue repair

## Abstract

Hepatectomy is an effective surgical method for the treatment of liver diseases, but intraoperative bleeding and postoperative liver function recovery are still key issues. This study aims to develop a composite hydrogel dressing with excellent hemostatic properties, biocompatibility, and ability to promote liver cell regeneration. The modified gelatin matrix (GelMA, 10%) was mixed with equal volumes of sodium alginate-dopamine (Alg-DA) at concentrations of 0.5%, 1%, and 2%. Then a cross-linking agent (0.1%) was added to prepare different composite hydrogels under UV light, named GelMA/Alg-DA-0.5, GelMA/Alg-DA-1 and GelMA/Alg-DA-2, respectively. All the prepared hydrogel has a porous structure with a porosity greater than 65%, and could be stabilized in a gel state after being cross-linked by ultraviolet light. Physicochemical characterization showed that the elastic modulus, water absorption, adhesion, and compressibility of the composite hydrogels were improved with increasing Alg-DA content. Furthermore, the prepared hydrogel exhibits *in vitro* degradability, excellent biocompatibility, and good hemostatic function. Among all tested groups, the group of GelMA/Alg-DA-1 hydrogel performed the best. To further enhance its application potential in the field of liver regeneration, adipose-derived mesenchymal stem cell exosomes (AD-MSC-Exo) were loaded into GelMA/Alg-DA-1 hydrogel. Under the same conditions, GelMA/Alg-DA-1/Exo promoted cell proliferation and migration more effectively than hydrogels without extracellular vesicles. In conclusion, the prepared GelMA/Alg-DA-1 composite hydrogel loaded with AD-MSC-Exo has great application potential in liver wound hemostasis and liver regeneration.

## 1 Introduction

Hepatectomy is an operation to remove local lesions in the liver, including liver tumors, liver trauma, liver abscess, and intrahepatic bile duct stones, etc (Quan et al., 1996; Chang et al., 2006; Longmire and Tompkins, 2012; Graham et al., 2018; Dilek et al., 2020). Although the liver itself has a regenerative function, patients suffering from diseases such as fatty liver or cirrhosis can severely affect postoperative liver function compensation and liver regeneration. Such patients are prone to postoperative complications (e.g., liver failure), which are life-threatening (Helling, 2006; Golse et al., 2013; Goh et al., 2018; Fagenson et al., 2021). In addition, due to hepatic insufficiency and coagulation dysfunction in patients with liver disease, it may be difficult to hemostasis the surgical wound, prolonging the operation time and affecting the operation quality. At present, hemostasis methods for hepatectomy include electrocoagulation, ultrasonic knife, suture hemostasis, etc. Suturing is a commonly used method of hemostasis in surgical operations, which is suitable for vascular bleeding of large vessels or vital organs. However, the ligature in suturing hemostasis is a foreign body, and there is a risk of rejection if it exists in the tissue for a long time (May 1991; Ruszczak and Schwartz, 2000). Intraoperative instrumental hemostasis (electrocoagulation and ultrasound knife) is more convenient than suture hemostasis, but may cause damage and embolization of adjacent vessels. Since most hemostatic devices are used at temperatures above 60°C, improper operation may cause irreversible damage to adjacent normal tissues (Sankaranarayanan et al., 2013; Vanella et al., 2016). Therefore, a new wound treatment strategy with good hemostatic effects, excellent biocompatibility, and promoting hepatocyte regeneration has important clinical application value for the recovery of liver function after hepatectomy.

Extracellular vehicles (EVs), i.e., exosomes, are lipid bilayer-bound vesicles secreted by cells that can carry various types of cargo between cells, such as proteases (matrix metalloproteinases, MMPs), inflammatory cytokines, growth factors, mRNA, miRNA, mitochondrial DNA, and genomic DNA (Buzas et al., 2014; Stremersch et al., 2016; Vader et al., 2016; Teng and Fussenegger, 2021). Exosomes vary in origin, size, content, and function (Dilsiz, 2020; Kalluri and LeBleu, 2020). For example, adipose-derived mesenchymal stem cells exosomes (AD-MSC-EXOs) have the functions of anti-inflammation, anti-apoptosis, promotion of tissue regeneration, and promotion of angiogenesis, etc., (Cai and Guo, 2023). Several studies have shown that exosomes derived from either mesenchymal stem cells or adipose stem cells have good repair and regeneration effects on various organ injury models (Yu et al., 2014; Hong et al., 2019; Tsiapalis and O’Driscoll, 2020; Cai and Guo, 2023). How to realize the local delivery of exosomes to lesion areas and prolong their functional time in these areas are the key issues in the use of exosomes for disease treatment.

The development of novel biomaterials has revolutionized the field of medicine, providing new solutions to a wide range of clinical problems. Hydrogel is a biocompatible material with three-dimensional structure, which is an excellent substitute for natural extracellular matrix (ECM). Due to their biocompatibility and biodegradable properties, hydrogels can be used as sustained-release drug carriers to deliver therapeutic agents (e.g., drugs, molecules, cells, etc.) directly at the damaged site (Peppas and science, 1997; Hoare and Kohane, 2008; Sun et al., 2019). Cheng et al. used gelatin methacrylate (GelMA) hydrogel-loaded bone marrow mesenchymal stem cell-derived exosomes for minimally invasive treatment of spinal cord injury (Cheng et al., 2021). Their findings showed that the prepared GelMA-based hydrogel enhanced the retention of exosomes, promoted the differentiation and extension of neurons, and attenuated the glial scar in damaged lesions (Cheng et al., 2021). Adhesion and regenerative properties of biomaterials at the application site are important factors for their ability to successfully regenerate damaged tissues. It has been reported that dopamine-modified hydrogels exhibited strong adhesion to tissues in the presence of blood or saliva (Wang et al., 2019; Han et al., 2022). Liu et al., used dopamine as an additive to improve the bulk mechanical and adhesive properties of the four-armed poly (ethylene glycol) (PEG-D4)-based Laponite injectable hydrogel, which has great application potential in tissue and wound repair. (Liu et al., 2014).

In this study, we designed a photo-crosslinked hydrogel capable of loading exosomes, with good adhesion, effective hemostasis, and excellent biocompatibility to promote liver cell regeneration. The photo-crosslinked hydrogels have the advantages of fast gelation, ease of use, and the ability to adapt to wounds of different shapes. The prepared hydrogel is based on GelMA with the addition of alginate modified with dopamine (Alg-DA) to increase adhesive properties, and then loaded with adipose-derived mesenchymal stem cells exosomes (ADMSCs-Exo) to promote cell proliferation and tissue regeneration. The physicochemical properties, hemostatic properties, biocompatibility, and cell proliferation of the prepared hydrogels were evaluated as well.

## 2 Materials and methods

### 2.1 Materials

All chemicals and reagents were of chemical grade and used without further purification. Gelatin (Gel), derived from cold-water fish skin, was purchased from Sigma-Aldrich (Shanghai, China). Dopamine (4-(2-Aminoethyl) benzene-1,2-diol, DA, 98%), sodium alginate, analytically pure, methacrylic anhydride (Methacrylic anhydride, MA), 94%, containing 0.2% topanol stabilizer, lithium phenyl-2,4,6-trimethylbenzoylphosphinate (LAP), ≥98%, and other reagents were purchased from McLean (Shanghai, China). The Cell counting Kit 8 (CCK8) was purchased from Shanghai Biyuntian Biotechnology Co., Ltd (Shanghai, China). The other reagents, culture medium and fetal bovine serum were purchased from Gibco-BRL (Burlington, Canada).

### 2.2 Preparation of GelMA/Alg-DA-based hydrogels

Gelatin (Gel) was added into the PBS solution, stirring in a warm water bath at 50°C for 1 h until it was completely dissolved, to prepare a 10% (w/v) Gel solution. MA was gradually added dropwise into the solution at a rate of 0.5 mL/min, then stirring for 1 h. The resultant (GELMA) was placed in a dialysis bag, dialyzed, filtered, and stored in a refrigerator at 4°C.

The preparation of dopamine-modified alginate (Alg-DA) was as follows. Briefly, alginate (Alg) was oxidized and purified, and recovered via freeze-drying. The synthesis of Alg-DA was synthesized by activating the carboxyl group on Alg and the amino group on DA. First, alginate (0.75% w/v) was dissolved in a 100 mM MES solution at a pH of 6. The entire dissolution process was carried out under anaerobic conditions by continuously supplying nitrogen. Then, 1-(3-dimethylaminopropyl)-3-ethylcarbodiimide (EDC) and N-hydroxysuccinimide (NHS) were dissolved in the 2-Morpholinoethanesulphonic acid (MES) solution, and subsequently added to the Alg solution. After stirring and mixing for 15 min, DA (0.95% W/V) was dissolved in the MES solution, and then added to the obtained Alg solution for reaction and stirred for 3 h. The molar ratio of NHS: EDC: DA was 1.2:3:1. After the reaction was completed, the mixed solution was placed in a dialysis bag with a molecular weight cut-off of 6–8 kDa, and dialyzed in PBS solution for 3 days. The resultant was concentrated at 800 g for 15 min in a 10-kDa centrifuge. Finally, the mixture was lyophilized to obtain the dopamine-modified alginate complex (Alg-DA). Alg-DA solutions (0.5%, 1%, or 2%) were mix with GelMA (10%), respectively at a volume ratio of 1:1. The photo-crosslinking agent LAP (0.1%) were added into the obtained series of solutions and mixed them well to form GelMA/Alg-DA composite hydrogels under ultraviolet light irradiation, namely, GelMA/Alg-DA-0.5, GelMA/Alg-DA-1, GelMA/Alg-DA-2. Following the above conditions, pure GelMA hydrogel was prepared as a control.

### 2.3 Characterizations of GelMA/Alg-DA-based hydrogels

#### 2.3.1 Infrared detection

Samples (Alg, DA, and Alg-DA) were analyzed by infrared detection using an infrared instrument (VERTEX70, Bruker, Germany). First, a sample (3–5 mg) and an appropriate amount of dry potassium bromide powder (mass ratio is about 5%) were placed in an agate mortar, grinded and mixed thoroughly. The obtained fine sample powder was applied for tableting (vacuum pressure 20 mmHg, pressed for 5 min). A Fourier transform infrared spectrometer was used for detection, and the scanning range was set 4,000–500 cm^−1^.

#### 2.3.2 Determination of pore size, and porosity

GelMA, GelMA/Alg-DA-0.5, GelMA/Alg-DA-1, GelMA/Alg-DA-2 hydrogels were lyophilized and pasted on the copper platform respectively. After spraying gold, the samples were observed under a scanning electron microscope (S3400N, Hitachi, Japan). The average pore size and porosity of the hydrogels were calculated with Nano measure software.

#### 2.3.3 Rheological measurements

The rheological properties of the GelMA/Alg-DA-based hydrogels were measured using a rheometer with a stainless steel parallel-plate rotor with a diameter of 25 mm (MCR 52 Instruments). G′ represents the elastic modulus of the sample, and G″ represents the viscous modulus of the sample. A dynamic strain scan (0.1–10 rad/s) was performed at room temperature to determine the linear viscoelastic range of the hydrogel, and the storage modulus (G ′) and loss modulus (G″) curves were recorded.

#### 2.3.4 Swelling analysis

The weight of the initial hydrogel sample (i.e., GelMA, GelMA/Alg-DA-0.5, GelMA/Alg-DA-1, or GelMA/Alg-DA-2) was record as M0. A sample was soaked into PBS buffer (pH = 7.4) at room temperature for 24 h. Then the hydrogel sample was taking out, and the moisture on its surface was quickly blotted dry with slightly damp filter paper. The weight of the hydrogel sample (i.e., the weight of the hydrogel after absorbing water) was recorded as M1. Calculate the hydrogel swelling ratio according to the formula:

$$Swelling\;ratio=\frac{M1-M0}{M0}\times 100\%$$

#### 2.3.5 Adhesion properties

The ability of the hydrogel to adhere to tissue was tested using fresh pig skin. A pigskin was cut into strips of 10 mm*30 mm, soaked and preserved in PBS solution. After wiping the wet surface of the pigskin, hydrogel solution sample was placed on its surface, and then covered with another piece of pigskin coated by a layer of the same hydrogel sample. The pigskins with hydrogel layers were exposed to ultraviolet light for 1 min. The bonding area was set to 10 mm*10 mm. The bonded pigskin was left at room temperature for 2 h, followed by the bond test. Adhesive properties were tested by lap shear testing at a speed of 2 mm/min using a material testing system (MTS Criterion 43, MTS Criterion). All tests were performed in triplicate to ensure the reliability and reproducibility of the results.

#### 2.3.6 Compression testing

The hydrogel samples were placed directly below a specialized gel strength probe (ELF3200, Bose, United States). The probe compressed the hydrogel until it ruptured, and the force required to break the hydrogel was recorded, defined as the gel strength. The test was performed with a maximum compression of 35%, at a compression rate of 0.05 mm/s.

#### 2.3.7 Mass loss analysis

A hydrogel sample was freeze-dried, weighed, and recorded as W0. Then the sample was soaked in a PBS solution containing 1000 U/ml lysozyme, and placed on a constant temperature shaker (37°C, 70 rpm). At the measurement time points (i.e., 3, 7, 14, and 21 days), the hydrogel sample was taken out, freeze-dried, weighted, and recorded as (Wt). The *in vitro* remaining mass ratio of hydrogel sample was calculated according to the formula:

$$Remaning\;mass\;\%=\frac{Wt}{W0}\times 100\%$$

### 2.4 Coagulation evaluation

The hydrogel sample was prepared into a cylinder with a size of 1.0*1.0*0.3 cm^3^, then placed in a polystyrene Petri dish at 37°C for 5 min, to simulate the internal environment of humans. A blood sample of 100.0 µL was added dropwise on the surface of the hydrogel until completely covered. The sample of hydrogel with blood were incubate at temperature 37°C for 5 min.

Subsequently, 5.0 mL of deionized water was added to the hydrogel sample, and the plate was shaken at 50 rpm for 10 min. The absorbance of the obtained supernatant (A_sample_) was detected with a spectrophotometer, and the detection wavelength was 540 nm. At the same time, dilute 100 µL of blood into 5 mL of deionized water, and its absorbance was used as a reference (A_control_). The blood clotting index (BCI) of the hydrogel was calculated using the following formula.

$$BCI\;\%=\frac{A_{sample}}{A_{control}}\times 100\%$$

### 2.5 Hemolysis evaluation

A hydrogel sample was added into a tube with sodium chloride injection (10 mL). For the Control groups, 10 mL of PBS (negative control, NC) and Triton solution (positive control, PC) was added to the tube. All the test tubes were kept in a constant temperature water bath (37°C) for 30 min, and then diluted rabbit blood was added to each test tube, and mix gently. These test tubes were incubated at 37°C for 60 min, then subjected to centrifugation at 800 g for 5 min. The absorbance of the supernatant was measured by spectrophotometry at a wavelength of 545 nm. Three tubes were prepared in parallel for measurements. The hemolysis ratio of the hydrogel was calculated using the following formula.

$$Hemolysis\;\%=\frac{A_{sample}-A_{NC}}{A_{PC}-A_{NC}}\times 100\%$$

### 2.6 Extraction and identification of exosomes derived from adipose-derived mesenchymal stem cells

Human adipose-derived mesenchymal stem cells (ADMSCs) from the third-generation rats were selected, and when the cells proliferated by 70%, the complete medium was replaced with a serum-free medium. After 48 h of starvation culture, the cell supernatant was collected in a 50 mL centrifuge tube. The sample was then subjected to centrifugation method (1,000 g/min for 10 min, 3 cycles) to extract exosomes. After centrifugation, the exosomes aggregated into a pellet at the bottom of the ultracentrifuge tube, and the supernatant was removed. Resuspend with pre-cooled PBS at 4°C, and ultracentrifuge at 100,000 g for 70 min to remove medium components. The obtained exosome pellet was resuspended with PBS and kept at −80°C for later use. BCA assay was used to determine the protein concentration of the exosomes. The morphology and particle size of exosomes were studied by TEM and DLS, respectively. Exosomes from ADMSCs were identified by Western Blot assay.

### 2.7 Preparation of GelMA/Alg-DA-1/Exo hydrogel

The GelMA/Alg-DA-1/Exo hydrogel was prepared by mixing a 1% Alg-DA solution with a 10% GelMA solution at a 1:1 volume ratio, and the extracted exosomes were added at a concentration of 100 μg/mL. The photoinitiator LAP was then added at a concentration equivalent to 0.1% of the GelMA mass concentration. The mixture was mixed thoroughly, and then exposed to a 405 nm, 3 W light source for 20 s to obtain the GelMA/Alg-DA-1/Exo hydrogel. The GelMA/Exo hydrogel was prepared following the same procedure.

### 2.8 The sustained-release exosomes profiles of the prepared composite hydrogels

Enzyme-linked immunosorbent assay (ELISA) was used to detect the sustained release of exosomes from the prepared hydrogels. 1 mL of hydrogel were added into a tube (50 mL) containing 10 mL of PBS buffer. PBS solution was collected at specific time points. The amount of PBS collected each time was 0.5 mL, and an equal amount of fresh PBS was replenished. ELISA kit was used to measure the exosomes content in the collected PBS solution.

### 2.8 Cell viability

The components of the hydrogel were dissolved and then sterilized by filtration with a Millipore syringe filter (0.22 μM). A hydrogel solution (500 μL) was pipetted into a 12-well plate, and illuminated with a 405 nm, 3 W UV light source for 20 s. After the hydrogel was fully solidified, culture medium with 10% of fetal bovine serum (FBS) and 1% penicillin/streptomycin containing either L929 cells (fibroblast) or LO2 cells (normal human hepatocyte) (2 × 10^4^ cells per well), were added and incubated continuously for 2 days. Cell viability analysis was performed at 24 h and 48 h after co-culture. The well plate was taken out at selected time point, washed twice with PBS solution. The hydrogel in which the cells were loaded was removed and finely minced. Then the mixture was centrifuged, and the supernatant was mixed with CCK8 solutions, followed by incubation at 37°C for 2 h. The absorbance (OD) of samples at a wavelength of 450 nm was measured using a microplate reader, and calculate the cell viability according to the formula as follows.

$$Cell\;Viablility\;\%=\frac{{OD}_{sample}}{{OD}_{control}}\times 100\%$$

### 2.9 Wound healing assay

LO2 cells were seeded in 24-well plates (2 × 1 ^4^ cells per well) to form a confluent monolayer. After culturing in FBS-free medium for 24 h, the monolayer cells were scratched with a 200 μlL tube tip to simulate an incision wound. Cells were then washed with PBS to remove cell debris, treated with hydrogel extract, and incubated at 37°C with DAEM medium containing 1% FBS. At desired time intervals (0 h, 12 h, 24 h), cell migration pattern was photographed.

$$Invaded\;area\;\%=\frac{A_{0}-A_{t}}{A_{0}}\times 100\%$$

Where A_0_ is the scratch area at 0 h; A_t_ is the area of the scratch area that cell migrated at different time points.

### 2.10 Statistical analysis

All data were expressed as mean ± standard deviation (SD). Statistical significance was carried out via one-way ANOVA with a Tukey *post hoc* test by SPSS. Statistical significance was noted as follows: *p < 0.05 and**p < 0.01.

## 3 Results and discussion

**FIGURE 1:**
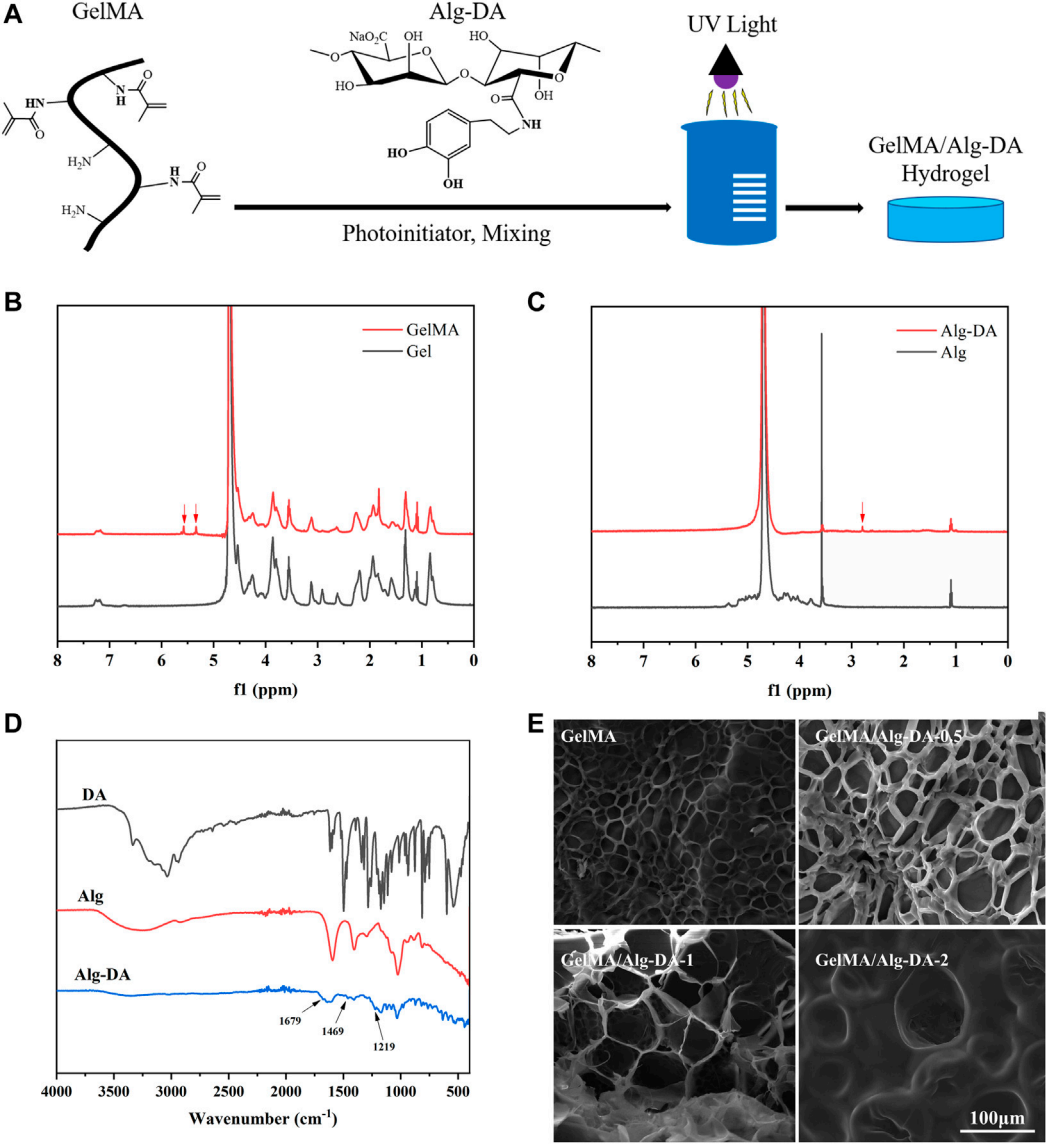
Synthesis and characterization of GelMA/Alg-DA-based hydrogels. **(A)** Scheme of the preparation of GelMA/Alg-DA hydrogels; **(B)** NMR spectra of Gel and GelMA; **(C)** NMR spectra of Alg and Alg-DA; **(D)** FTIR spectra of Alg, DA and Alg-DA; **(E)** SEM pictures of GelMA and GelMA/Alg-DA hydrogels, scale bar 100 μm.

In this study, GelMA/Alg-DA hydrogels were prepared through a series of reactions, as illustrated in Figure 1A. First, gelatin was modified to obtain GelMA as described in the Method section. Then sodium alginate was reacted with dopamine to prepare Alg-DA. Under the action of photo-initiator LAP and ultraviolet light, the obtained product was mixed with GelMA in different proportions to obtain a series of photo-crosslinked hydrogels GelMA/Alg-DA. The photocuring time of the prepared hydrogels were listed in Supplementary Table S1. Comparative nuclear magnetic resonance (NMR) analysis revealed the presence of the characteristic functional groups of methacrylic anhydride (*δ* = 5.3 and 5.5 ppm) in GelMA (Figure 1B), thereby confirming the successful synthesis of GelMA. Figure 1C displayed the characteristic functional group of dopamine (*δ* = 2.8) in Alg-DA, further verifying the successful preparation of Alg-DA. Fourier-transform infrared (FTIR) spectroscopy was employed to investigate the successful modification of sodium alginate with dopamine, specifically to ascertain whether Alg-DA was successfully prepared. As shown in Figure 1D, compared with Alg and DA, three new characteristic peaks appeared in the spectrum of Alg-DA. They were amide I band (1,679 cm^-1^), amide II band (1,469 cm^-1^) and amide III band (1,219 cm^-1^), respectively. This was caused by the reaction of sodium alginate and dopamine to form amide bond C=O, in which 1,679 cm^-1^ was the stretching vibration absorption peak of amide bond C=O bond. The spectral results showed that dopamine successfully modified sodium alginate to form Alg-DA. The microstructure of the hydrogel was characterized by SEM (Figure 1E). These four hydrogel groups (GelMA, GelMA/Alg-DA-0.5, GelMA/Alg-DA-1, and GelMA/Alg-DA-2) were all porous structures with a porosity greater than 65% (Table.1). Compared with the other three hydrogel groups, GelMA hydrogel had the smallest pore size (27.35 ± 6.73 μm), and the pores of GelMA hydrogel were not interconnected. As the viscosity of the system increased with the content of Alg-DM, the pore size of the GelMA/Alg-DM hydrogel increased gradually. The pore sizes of GelMA/Alg-DM-0.5, GelMA/Alg-DM-1 and GelMA/Alg-DM-2 were 56.76 ± 11.17 μm, 67.15 ± 9.61 μm and 78.26 ± 11.86 μm, respectively. When the addition of Alg-DM increased to 2%, the pore size of the hydrogel GelMA-Alg-DM-2 group became larger and most of them were closed, and the pores were not connected. The porosity of this group was 66.57% ± 3.79%, which was the lowest porosity among the four hydrogel groups. This might be due to the fact that the addition of excessive Alg-DM would increase the viscosity of the system and form a tighter hydrogel structure, resulting in a lower porosity of the hydrogel. By comparing the pore size and porosity, it could be concluded that the pore size and porosity of GelMA-Alg-DM-0.5 and GelMA-Alg-DM-1 were the most suitable among the four prepared hydrogel groups.

**TABLE 1:** The porosity and pore size of prepared hydrogels.

Hydrogel	Pore size (μm)	Porosity (%)
GelMA	27.35 ± 6.73	74.07 ± 4.99
GelMA/Alg-DA-0.5	56.76 ± 11.17	71.74 ± 3.18
GelMA/Alg-DA-1	67.15 ± 9.61	82.48 ± 2.31
GelMA/Alg-DA-2	78.26 ± 11.86	66.57 ± 3.79

This network structure of hydrogels helps to moisten the moist environment at the wound site. In addition, the pores are interconnected with each other, and the cells can migrate and grow well within the hydrogel. The porous structure of prepared hydrogel would promote the transmission of nutrients within the cells, and provide conditions for the rapid healing of different wound tissues (Sun et al., 2018; Huang et al., 2020).

**FIGURE 2:**
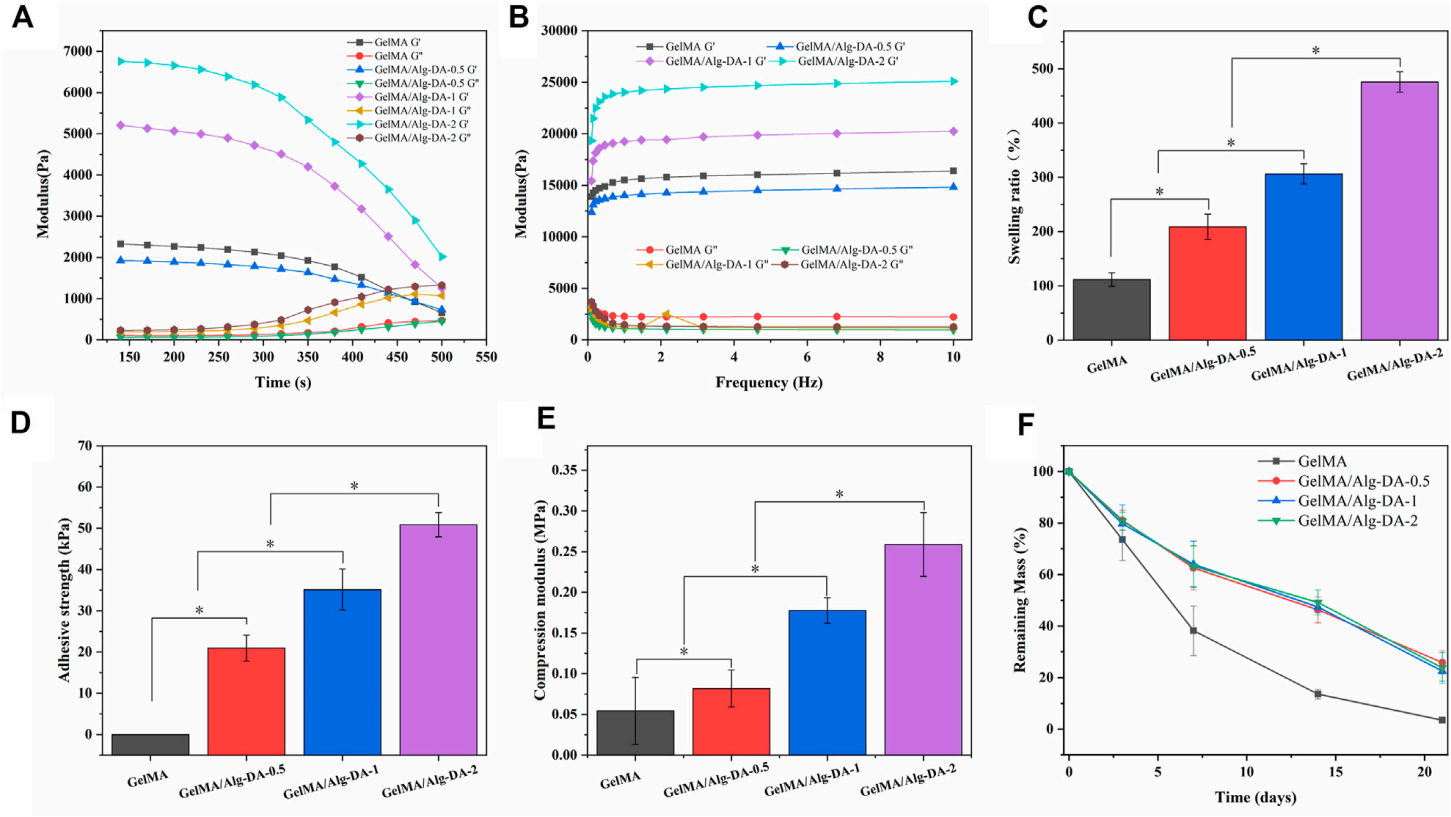
Characterization of GelMA/Alg-DA-based hydrogels: **(A)** Time-dependent rheology graph showing rheological properties of the prepared hydrogels over time. **(B)** Frequency-dependent rheology graph showing rheological properties of the prepared hydrogels as a function of frequency. **(C)** Swelling ratio of the prepared hydrogels. **(D)** Adhesive strengths of the prepared hydrogels. **(E)** Compression properties of the prepared hydrogels. **(F)** In vitro degradation behaviors of the prepared hydrogels.

The interaction inside the hydrogel is one of the key factors affecting cell growth or drug release, and the change of the internal structure of the hydrogel is closely related to the rheological properties of its matrix material. In rheology, the elastic modulus (G′) characterizes the solid-like behavior of a material, and the viscous modulus (G″) characterizes the liquid-like behavior of the material. When the elastic modulus of the hydrogel is greater than the viscous modulus, the hydrogel behaves as a gel state. The elastic moduli (G′) and viscous moduli (G″) of the four hydrogels as a function of time and frequency are shown in Figures 2A,B, respectively. In Figure 2A, when the four hydrogels were gelled, G′ was always greater than G″ as time increased, indicating that the hydrogels could be stabilized in a gel state after UV curing. With the increase of Alg-DM content, the elastic modulus of the hydrogel continuously increased, indicating that the addition of Alg-DM would increase the stiffness of the hydrogel. In Figure 2B, it showed that the G′ values for all four hydrogel materials are greater than their corresponding G″ values. This finding indicated that the prepared hydrogels could stably maintain their solid state within the tested frequency range.

The good water absorption capacity of hydrogel enables it to keep the environment moist, which is essential for better wound repair (Dhivya et al., 2015; Gupta et al., 2019; Xiang et al., 2020). The swelling properties of the GelMA/Alg-DA-based hydrogels were shown in Figure 2C. All the prepared hydrogel could absorb more than 1 times the total amount of its own solution. The water absorption capacity of GelMA hydrogel was the lowest among the four groups, and its swelling ratio was 103.8% ± 14.8%. With the addition and content of Alg-DA, the water absorption ability of GelMA/Alg-DA system hydrogel has been greatly improved, the swelling ratios of GelMA/Alg-DA-0.5, GelMA/Alg-DA-1 and GelMA/Alg-DA-2 were 210.4% ± 27.2%, 305.5% ± 17.5%, 460% ± 12.3%, respectively. This result indicated that the prepared GelMA/Alg-DM hydrogels can greatly absorb the exudate of the wound, maintain the moist environment of the wound, and be beneficial to the repair and healing of the wound.

For the hemostasis and repair of wounds, a key factor is the successful long-term adhesion of the hydrogel to the wound, blocking the bleeding site and thus stopping the bleeding. In addition, the long-term adhesion and retention of hydrogel can keep the wound moist for a long time. Cells proliferate stably in the hydrogel medium, promoting wound repair and healing. Therefore, the adhesion, mechanical properties and appropriate degradation rate of hydrogels are very important factors affecting their application in biomedicine. The results showed that GelMA hydrogel has little adhesive properties and the compressive modulus is 0.05 MPa (Figures 2D,E). The increase of Alg-DA content improved the adhesion properties of GelMA/Alg-DA-based hydrogels, as the adhesion of GelMA/Alg-DA-0.5, GelMA/Alg-DA-,1 and GelMA/Alg-DA-2 were 20.05 ± 3.85 KPa, 35.05 ± 4.75 KPa, and 50.05 ± 2.85 KPa, respectively. In addition, with the increase of Alg-DA content, the compressive modulus of GelMA/Alg-DA hydrogel was also enhanced.

To analyze the degradation of prepared hydrogels *in vitro*, GelMA/Alg-DA-based hydrogel were soaked in PBS solution containing 1000 U/ml lysozyme (Figure 2F). On the 21st day, the remaining mass of GelMA was 3.46% ± 1.20%; while the remaining mass of the other three groups was almost the same, around 20%. The addition of Alg-DA slowed down the degradation rate of GelMA-Alg-DM hydrogels and prolonged the use of GelMA/Alg-DA hydrogels. Compared with GelMA hydrogel, the hydrogels added with Alg-DA content showed greater compression performance and slower degradation rate. This might be explained by the following two points. First, the addition of Alg-DA increased the viscous of the hydrogel system. Secondly, the chemical crosslinking of Alg-DA and GelMA improved the compressibility of prepared hydrogel and makes it more stable during degradation.

**FIGURE 3:**
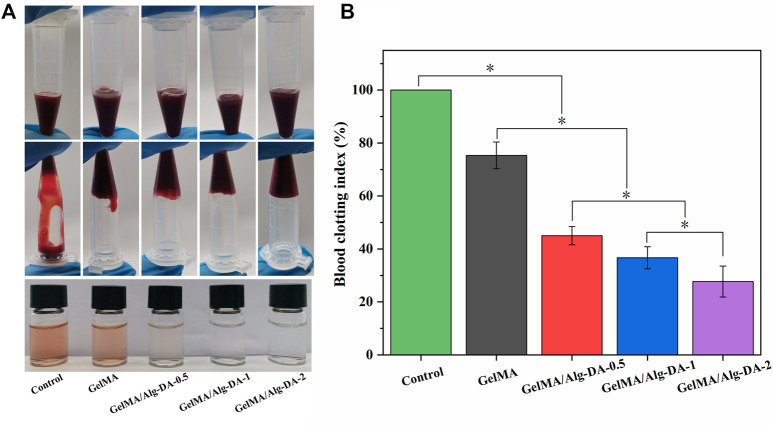
Hemostatic performance evaluation for the GelMA/Alg-DA-based hydrogels. **(A)** Photographs of blood coagulation treated with the prepared hydrogels; **(B)** Whole blood clotting index of the prepared hydrogels.

The prepared GelMA/Alg-DA-based hydrogel showed good tissue adhesion properties (Figure 2), and has a good advantage in hemostasis. In this study, the coagulation index (BCI) was used to evaluate the hemostatic effect of the prepared hydrogels (Figure 3). The lower the BCI, the better the coagulation effect. Compared with the blank group and the GelMA group, after 5min of free coagulation, the amount of uncoagulated blood in the other GelMA/Alg-DA-based groups were generally reduced, and the number of suspended blood cells visible to the naked eye was decreased (Figure 3A). BCI quantitative analysis showed the BCI of the prepared hydrogel decreased with the increase of Alg-DA content (Figure 3B). Coagulation experiments indicated that the prepared GelMA/Alg-DA hydrogels (i.e., GelMA/Alg-DA-0.5, GelMA/Alg-DA-1, GelMA/Alg-DA-2) have a good hemostatic effect.

**FIGURE 4:**
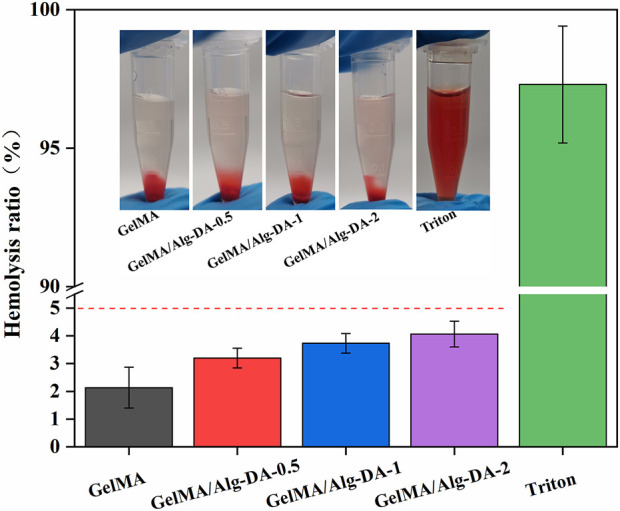
Hemolysis performance evaluation for the GelMA/Alg-DA-based hydrogels.

Biomaterials for clinical use should be evaluated for their potential hemolytic properties. Hemolysis (rupture of red blood cells) in the body can lead to anemia, jaundice, and other pathologic conditions. The results of hemolysis rate (Figure 4) showed that the hemolysis rates of either GelMA or the GelMA/Alg-DM-based hydrogels were all less than 5%, indicating that the prepared hydrogels have good blood compatibility.

**FIGURE 5:**
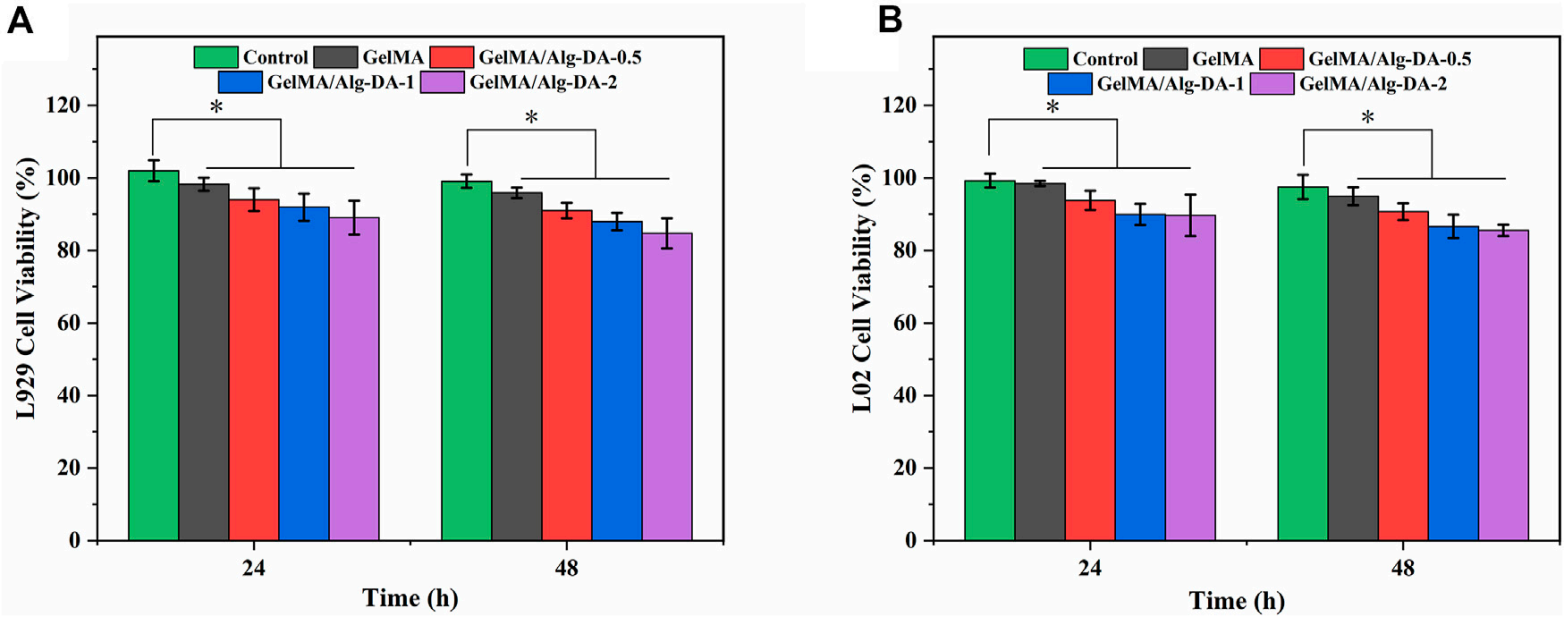
Cell viability of L929 **(A)** and LO2 **(B)** with the GelMA/Alg-DA-based hydrogels.

Cytotoxicity evaluation is one of the important steps in the clinical application of hemostatic agents and wound dressings. In this study, the survival rates of fibroblast L929 and normal human hepatocyte LO2 cells co-cultured with different hydrogel groups were evaluated using the CCK-8 assay (Figure 5). After incubation of 48 h, both L929 and LO2 cells grew well in the prepared four hydrogels, as the survival rates were all higher than 80%. This result demonstrated the good cytocompatibility of the prepared hydrogels. Although the cell survival rate of GelMA/Alg-DA-based hydrogels decreased slightly with the increase of Alg-DA content, the cell survival rates of these hydrogels were still greater than 80%, indicating that the GelMA/Alg-DA-based hydrogel was beneficial to cell proliferation. Based on the results of physical and chemical properties, hemostatic performance, hemolysis rate assay, and cytotoxicity, it showed that GelMA/Alg-DA-1 hydrogel group has the best performance among all groups, and could be used for subsequent cell function tests.

**FIGURE 6:**
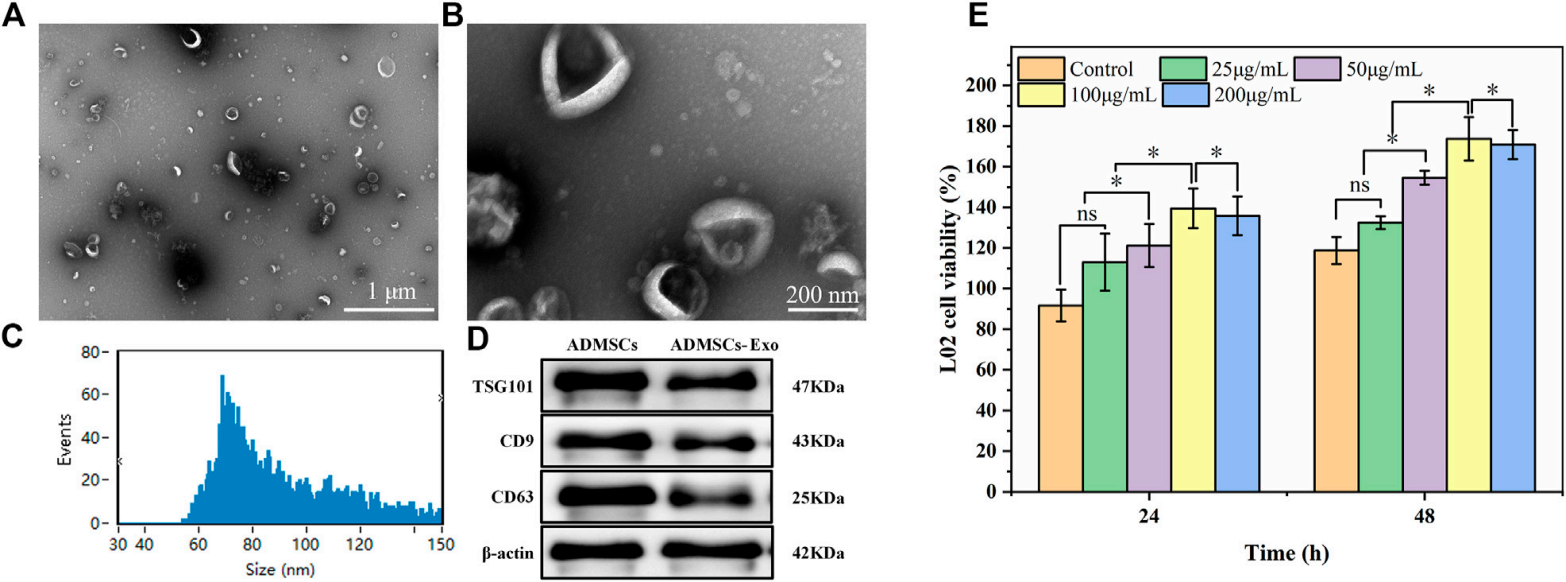
Characteristics of the ADMSCs-derived exosomes. Panels **(A)** and **(B)** referred to morphology of ADASCs-Exo. Panel **(C)** referred to particle size distribution of the Exo. Panel **(D)** refer to Western Blot assay of the Exo. **(E)** Cytotoxicity of the Exo at different concentrations on LO2 cells.

ADMSCs-Exo were extracted from adipose-derived mesenchymal stem cells, and identified by TEM pictures, particle size analysis, and WB assay. As shown in Figures 6A,B, most ADMSCs-Exo were oval vesicles. The particle size of the ADMSCs-Exo was concentrated in the range of 30–150 nm, with an average particle size of 89.43 ± 22.12 nm (Figure 6C). Western blot assay confirmed that the biomarkers TSG101, CD63, and CD81 were positive in the samples of ADMSCs and ADMSCs-Exo (Figure 6D). These results suggested the successful extraction of ADMSCs-Exo with a particle size of 89.43 ± 22.1 nm. CCK-8 assay was used to explore whether ADMSCs-Exo had a proliferation effect on LO2 cells (Figure 6E). After incubation of 48 h, ADMSCs-Exo showed a concentration-dependent effect on the proliferation of LO2 cells. In the concentration range of 25–100 μg/mL, as the concentration of ADMSCs-Exo increased, the survival rate of LO2 cells increased. However, when the concentration of ADMSCs-Exo was 200 μg/mL, its effect on cell proliferation was slightly lower than 100 μg/mL.

**FIGURE 7:**
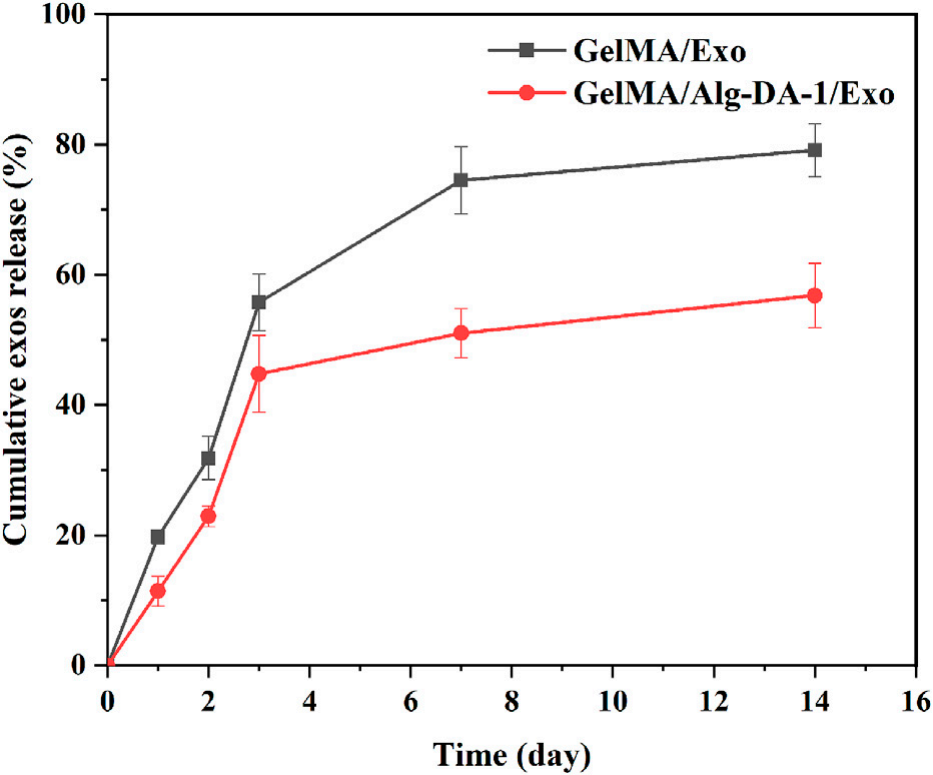
Cumulative release profiles of Exo from the GelMA/Exo and GelMA/Alg-DA-1/Exo hydrogels over 14 days.

ADMSCs-Exo could be released continuously for more than 14 days in the prepared GelMA/Exo and GelMA/Alg-DA-1/Exo hydrogels (Figure 7). At Day 14, the exosomes released from GelMA/Exo and GelMA/Alg-DA-1/Exo were 80% and 50%, respectively. In addition, the release rate of exosomes from GelMA/Alg-DA-1/Exo group was slower than that from GelMA/Exo group. This might be due to the high viscosity and high degree of cross-linking of the GelMA/Alg-DA-1/Exo hydrogel system, which prevented exosomes from being directly and suddenly released from the hydrogel. The sustained release behavior of GelMA-Alg-DM-1/Exo hydrogel exosomes indicated its great potential for liver repair and regeneration applications.

**FIGURE 8:**
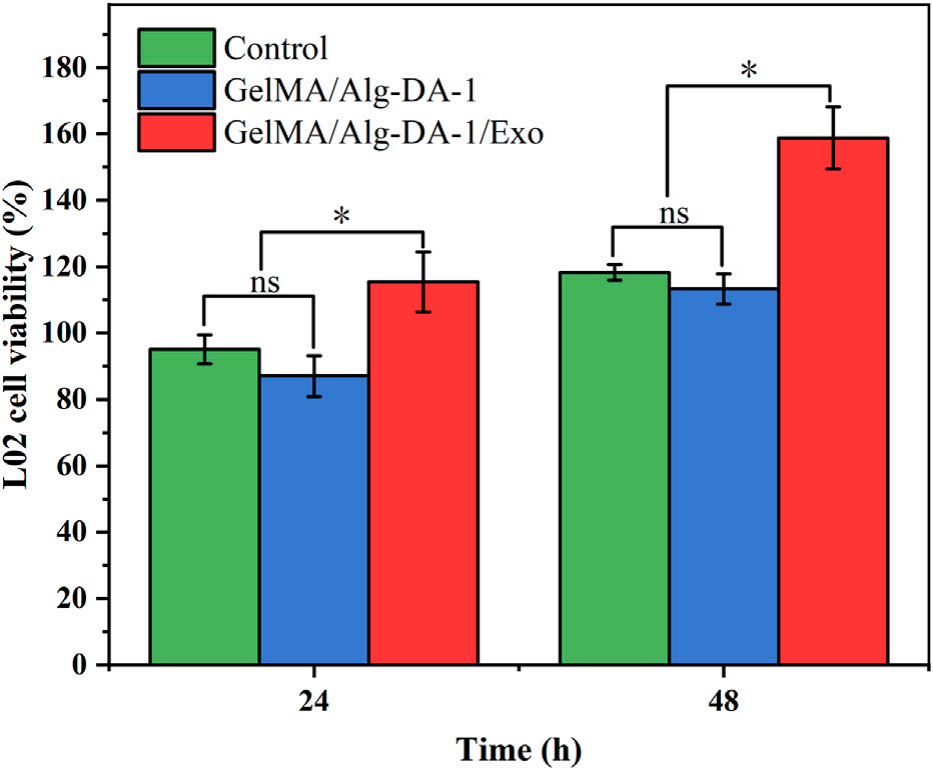
Cell viability of LO2 treated with various hydrogels over 48 h.

Exosomes could be used as an alternative to MSCs transplantation, thereby alleviating the safety issues and limitations associated with MSCs transplantation. Several studies have found that exosomes play important roles in cell proliferation and migration, angiogenesis, immune regulation, osteogenic differentiation, etc., and can also repair tissues damage (e.g., heart, liver, brain, kidney) (Shabbir et al., 2015; Hade et al., 2021; Huang et al., 2021). According to the experimental results (Figure 6D; Figure 7), we loaded ADMSCs-Exo into GelMA/Alg-DA-1 to ensure that the release amount was not lower than 100 μg/mL on the first day. The effect of exosomes released from the composite hydrogel on the proliferation of LO2 cells was investigated (Figure 8). After 48 h incubation, the cells treated with GelMA/Alg-DA-1/Exo hydrogels showed the highest survival rate, which was about 1.4-fold higher than that of the Control and GelMA/Alg-DA groups. This could be attributed to the release of exosomes from the hydrogel, which could substantially promote the cell proliferation under the same conditions.

**FIGURE 9:**
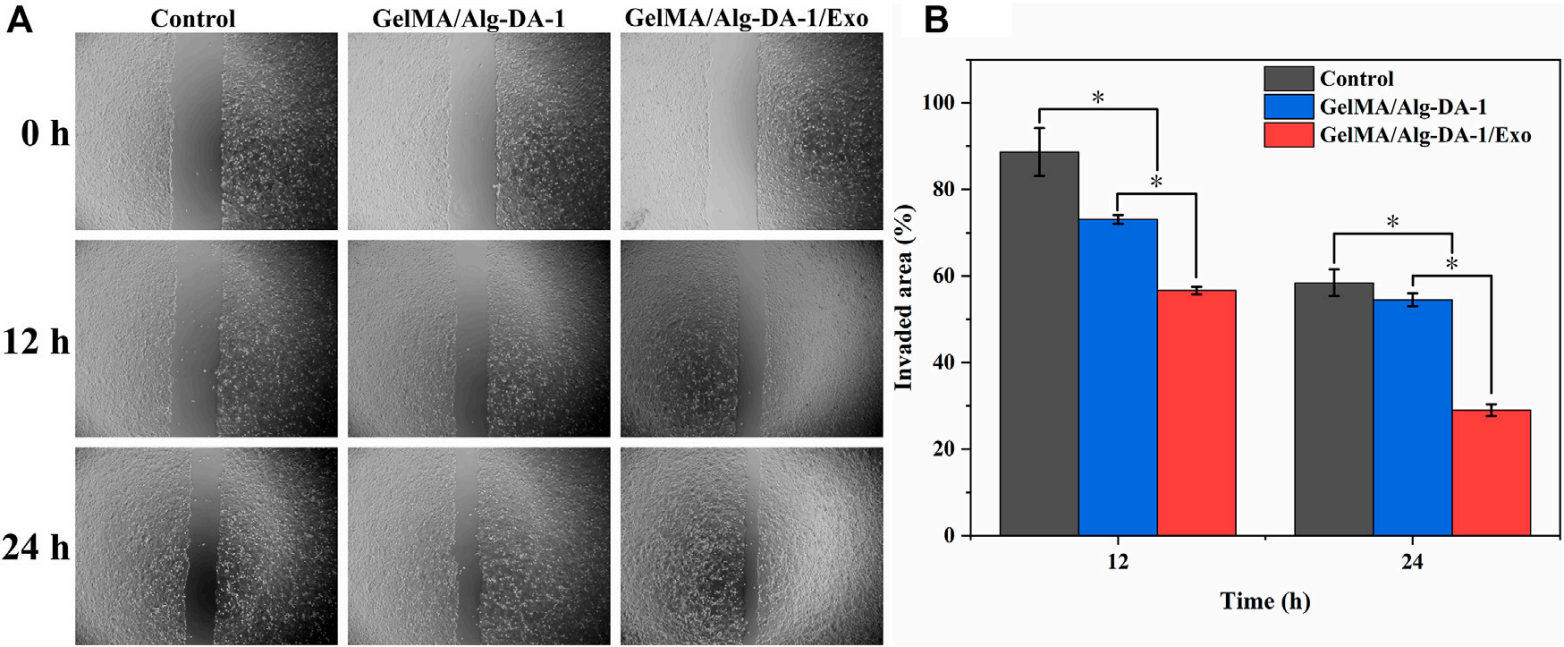
Wound healing assay to test migration ability of LO2 cells treated with the prepared hydrogels. **(A)** Representative pictures of the scratch area on 0, 12, 24 h after the cells treated different hydrogel groups. **(B)** Quantitative analysis of the relative scratch area of LO2 cells at different timepoints.

To investigate whether the prepared composite hydrogel affects the migration ability of LO2, a wound healing assay were performed (Figure 9). After scratch treatment for 12 h, LO2 cells treated with either GelMA/Alg-DA-1 or GelMA/Alg-DA-1/Exo groups, gradually grew towards the scratch. For the Control group, the migration area was almost equal to the original scratch area without significant change. After 24 h treatment, the scratch area of GelMA-Alg-DA-1/Exo group decreased significantly, which was significantly larger than that of Control and GelMA/Alg-DA-1 groups. These results were consistent with the results of CCK8 assay (Figure 8), indicating that GelMA-Alg-DM-1/Exo could promote the growth of LO2 cells and enhance their migration ability.

## 4 Conclusion

In this study, we modified gelatin and alginic acid, and then mixed them in a certain ratio to prepare adhesive GelMA/Alg-DA hydrogels under the action of photo-crosslinker LAP and UV light. Experimental results showed that the prepared hydrogels have a porous structure, good water absorption. With the addition of Alg/DA content increased, adhesion and compressibility of the GelMA/Alg-DA-based hydrogels enhanced. All the prepared hydrogels could be degraded *in vitro*, and they have hemostatic function and excellent biocompatibility, indicating their potential to be serve as hemostatic agents. Based on the results of physical and chemical properties, hemostatic performance, hemolysis rate assay, and cytotoxicity, it showed that GelMA/Alg-DA-1 hydrogel group has the best performance among all groups, and could be used for cell function tests. Therefore, ADMSCs-Exo were successfully extracted and loaded into GelMA/Alg-DA-1 hydrogel. The composite hydrogel sustainably released up to 50% of Exo over 14 days. Compared with the hydrogel without Exo, GelMA/Alg-DA-1/Exo could effectively promote the cell proliferation and migration ability under the same conditions. In summary, the prepared composite hydrogel loaded with AD-MSC-Exo has great application potential in liver wound hemostasis and liver regeneration.

## Data Availability

The raw data supporting the conclusion of this article will be made available by the authors, without undue reservation.
