# Supplementary material for: Photo-crosslinked adhesive hydrogel loaded with extracellular vesicles promoting hemostasis and liver regeneration
Source: Front Bioeng Biotechnol. 2023 May 10;11:1170212. doi: 10.3389/fbioe.2023.1170212 (PMC10208220; doi:10.3389/fbioe.2023.1170212)
Supplement: Supplementary file 1 [file Table1.docx]

| **GROUP** | **gel time 1** | **gel time 2** | **gel time 3** | **Average** | **StDev** |
| --- | --- | --- | --- | --- | --- |
| GelMA | 23.84 | 25.64 | 24.51 | 24.66 | 0.91 |
| GelMA/Alg-DM-0.5 | 24.01 | 26.11 | 25.33 | 25.15 | 1.06 |
| GelMA/Alg-DM-1 | 25.33 | 25.13 | 25.5 | 25.32 | 0.19 |
| GelMA/Alg-DM-2 | 26.15 | 25.61 | 25.49 | 25.75 | 0.35 |

Table S1 Photocuring time of different hydrogels
